# Marginally outer trapped tubes in de Sitter spacetime

**DOI:** 10.1007/s11005-024-01884-y

**Published:** 2024-12-13

**Authors:** Marc Mars, Carl Rossdeutscher, Walter Simon, Roland Steinbauer

**Affiliations:** 1 Departamento de Física Fundamental, Universidad de Salamanca, Plaza de la Merced s/n, 37008 Salamanca, Spain; 2 Fakultät für Mathematik, Universität Wien, Oskar-Morgenstern Platz 1, 1090 Vienna, Austria

**Keywords:** CMC surface, CMC foliation, de Sitter spacetime, Stability of marginally outer trapped surface, 53C42, 58J60, 58J70, 83C20

## Abstract

We prove two results which are relevant for constructing marginally outer trapped tubes (MOTTs) in de Sitter spacetime. The first one (Theorem 1) holds more generally, namely for spacetimes satisfying the null convergence condition and containing a timelike conformal Killing vector with a “temporal function”. We show that all marginally outer trapped surfaces (MOTSs) in such a spacetime are unstable. This prevents application of standard results on the propagation of stable MOTSs to MOTTs. On the other hand, it was shown recently, Charlton et al. (minimal surfaces and alternating multiple zetas, arXiv:2407.07130), that for every sufficiently high genus, there exists a smooth, complete family of CMC surfaces embedded in the round 3-sphere $\mathbb {S}^3$. This family connects a Lawson minimal surface with a doubly covered geodesic 2-sphere. We show (Theorem 2) by a simple scaling argument that this result translates to an existence proof for complete MOTTs with CMC sections in de Sitter spacetime. Moreover, the area of these sections increases strictly monotonically. We compare this result with an area law obtained before for holographic screens.

## Introduction

Our general setting is a smooth *n*-dimensional ($n \ge 3$), oriented and time-oriented Hausdorff manifold $(\mathcal{M}, g)$ with a smooth metric of signature $(-,+,...+)$. In Sect. 2, we require the Ricci tensor $\hbox {Ric} _g$ to satisfy the null convergence condition $\hbox {Ric} _g(\ell ,\ell ) \ge 0$ for all null vectors $\ell$, while in Sect. 3, we restrict ourselves to de Sitter spacetime (dS). We first recall well-known key definitions to also fix our notation. Note that we use the “physics” convention throughout, in particular in Definition 1 where most mathematics references (e.g. [11]) define the mean curvature by $\widehat{H} = H/\hbox {dim}~ \mathcal{U}$.

### Definition 1

Let $\mathcal{U}$ be a hypersurface (i.e. a submanifold of codimension one) embedded in any semi-Riemannian manifold $\mathcal{N}$. The **mean curvature** *H* of $\mathcal{U}$ is the trace of its second fundamental form.

### Definition 2

Let $\mathcal{F}$ be a spacelike surface of codimension two embedded in $\mathcal{M}$ with future directed, orthogonal null vectors $\ell ^{\pm }$, and denote the null expansions of the corresponding families of emanating geodesics by $\theta ^{\pm } = tr_\mathcal{F} (\nabla \ell ^{\pm })$.

- A **Marginally outer trapped surface (MOTS)** or just **Marginal surface** $\mathcal{F}$ is defined by $\theta ^{+} =0$. Here, $\ell ^+$ defines the “outer” direction; the latter is ill-defined for minimal surfaces for which $\theta ^{-} = 0$ as well. (As to terminology cf. Remark 3 in Sect. 2).
- A **Marginally trapped surface (MTS)** is a MOTS with either $\theta ^{-} < 0$ or $\theta ^{-} > 0$ (we avoid the term “past trapped” or “antitrapped” for the latter case).
- A **Marginally outer trapped tube (MOTT)** $\mathcal{T}$ is a hypersurface foliated by MOTS.
- A **Marginally trapped tube (MTT)** or **holographic screen** is a hypersurface foliated by MTSs.

MOTS, MTS or trapped surfaces which satisfy $\theta ^{+} < 0$ and $\theta ^{-} < 0$ are key in singularity theorems which yield incomplete geodesics. However, in a typical collapse scenario, there is not only the (outermost) “key MOTS”, but also a plethora of other, seemingly “spurious” MOTS (see e.g. [6, 22] and references therein). One motivation of this work is to better understand such spurious MOTS.

Apart from these collapse scenarios, MOTS are normally also abundant in non-singular spacetimes which do not satisfy all requirements for the singularity theorems. We mention two settings in which they are worth studying: Firstly, under additional assumptions, null geodesics emanating from MOTS have conjugate points while still being complete. This suggests a recent definition [21] of the “outward affine size” of a non-singular universe with a MOTS. Secondly, for asymptotically dS spacetimes, there are results on the “visibility” of future weakly trapped surfaces (defined by $\theta ^{\pm }\le 0$) from infinity [12].

A key property of MOTS is their (in)stability, cf. Definition 3 and Remark 2. Here, we recall important properties of strictly stable MOTSs: They have spherical topology, inherit all symmetries of the ambient space, and propagate along achronal MOTTs in a given spacelike foliation of spacetime [3, 4]. Somewhat weaker results hold in the non-strictly stable case. Moreover, stability also plays a role in a singularity theorem (cf. Theorem 7.1 and Remark 7.2 in [4]).

The present work has two somewhat independent aspects exposed in Sects. 2 and 3. In Sect. 2, we show the absence of stable MOTS in a class of spacetimes containing a timelike conformal Killing field with a “temporal function”. The precise result is as follows:

### Theorem 1

Let $(\mathcal{M},g)$ be an *n*-dimensional ($n \ge 3$) spacetime satisfying the null convergence condition. Assume $(\mathcal{M},g)$ admits a future directed timelike conformal Killing field $\xi$, i.e.

$$\begin{aligned} \pounds _{\xi } g = 2 \Psi g \end{aligned}$$

where $\pounds _{\xi }$ is the Lie derivative and $\Psi$ is a temporal function, i.e. its gradient is timelike and past directed. Then, $(\mathcal{M},g)$ admits no stable MOTS.

In Sect. 3, we restrict ourselves to dS for which $\hbox {Ric} _g = (n - 1) \delta ^2\,g$, $\delta$ a positive constant. It can be globally written in the form

1 $$\begin{aligned} g_{\tiny {\text {dS}}} = \frac{1}{\delta ^2 \cos ^2 \sigma } \left( - d \sigma ^2 + g_{\mathbb {S}^{n-1}} \right) \end{aligned}$$

where $\sigma \in \left( - \frac{\pi }{2}, \frac{\pi }{2} \right)$ is taken to increase to the future, and $g_{\mathbb {S}^{n-1}}$ is the standard metric on the round unit sphere $\mathbb {S}^{n-1}$.

Choosing $\xi = \frac{\partial }{\partial \sigma }$ in Theorem 1, we show in Corollary 1 that all MOTSs in dS are unstable. Hence, the above-said results of [3, 4] on topology, symmetry and evolution to achronal MOTTs do not apply. To analyse MOTTs, we now restrict ourselves to 3+1 dimensions and focus on umbilic and constant curvature slicings of dS (with curvatures of all possible signs). It is easy to show (Lemma 2 in Sect. 3.2.1) that in this setting, MOTSs are automatically MTSs and (as hypersurfaces within the slices) surfaces of constant mean curvature (CMC). This allows, in particular, constructing a MTSs from certain families of CMC surfaces in either flat $\mathbb {R}^3$, the hyperbolic space $\mathbb {H}^3$ or the round $\mathbb {S}^3$. We consider the former two settings (in Sects. 3.2.2 and 3.2.3) as a “warmup” and focus on $\mathbb {S}^3$ (cf. Sect. 3.2.4).

In Sect. 3.2.4, we analyse, and then dS in terms of its “standard” umbilic slicing (1) by round three spheres ${\mathbb S}^3_{\sigma }$ of radius $\rho = \delta ^{-1} \cos ^{-1} \sigma$. In this slicing, MOTS with $\theta ^{\pm } = 0$ correspond to CMC surfaces with mean curvature $H = \mp 2 \delta \sin \sigma$, thus in particular minimal for $\sigma = 0$. The resulting MTSs are easily found explicitly in the case of spherical and toroidal MTS sections. In the former case, the MTSs are null surfaces with sections of constant area, while in the latter case, the MTSs are timelike and their area increases monotonically from the Clifford torus at $\sigma = 0$ to $\sigma \rightarrow \pm \frac{\pi }{2}$ (where it diverges).

On the other hand, knowledge of CMC surfaces of genus ${\mathfrak g}$ larger than one in $\mathbb {S}^3$ is rather rudimentary. However, for every ${\mathfrak g}$ sufficiently large (${\mathfrak g} \gg 1$), Charlton et al. [11] recently proved existence of a complete family of CMC surfaces $f_{\psi }^{\mathfrak g}$, $\big (\psi \in \big (-\frac{\pi }{4}, \frac{\pi }{4}\big )\big )$ embedded in the unit sphere $\mathbb {S}^3$. This family connects the well-known Lawson minimal surface $\xi _{1,{\mathfrak g}}$ (for $\psi = 0$) with a doubly covered geodesic sphere (for $\psi = \pm \frac{\pi }{4}$). Moreover, these authors show that the Willmore energy of these surfaces, defined by

2 $$\begin{aligned} W = A \Big (\frac{H^2}{4} + 1\Big ), \end{aligned}$$

where *A* is the area, increases strictly monotonically from the Lawson surfaces towards the limiting spheres.

After quoting and slightly extending the results of [11] as Theorem 3 in Sect. 3, we describe its straightforward application to dS. In fact, we only need to rescale the geometry from the $\mathbb {S}^3$ of unit radius to the spherical sections of dS whose radius blows up with the cosmological time $\sigma$ as sketched above. Remarkably, this scaling entails that the Willmore energy of the CMC surfaces found in [11] translates just to the area of the MTS sections in the dS setting and yields the corresponding monotonicity statement. Thus, our adaptation of the results of [11] reads as follows.

### Theorem 2

Our setting is de Sitter spacetime in coordinates (1) for $n = 4$. For every ${\mathfrak g} \in \mathbb {N}$, ${\mathfrak g} \gg 1$, there exist a smooth function $\sigma : (-\frac{\pi }{4}, \frac{\pi }{4})\ni \psi \mapsto \sigma (\psi ) \in (-\sigma _{m}, \sigma _{m})$ and a smooth family of conformal CMC embeddings $\mathcal{F}_{\psi }^{\mathfrak g}: \mathcal{R}^{\mathfrak g} \rightarrow \mathbb {S}^3_{\sigma }$ of a Riemann surface $\mathcal{R}^{\mathfrak g}$ of genus ${\mathfrak g}$ into the round 3-sphere $\mathbb {S}^3_{\sigma }$ of radius $\rho _{\sigma } = \delta ^{-1} \cos ^{-1}\sigma$ such that

1. $\mathcal{F}_{0}^{\mathfrak g}$ is the Lawson surface $\xi _{1,{\mathfrak g}}$ of genus ${\mathfrak g}$.
2. For $\psi \rightarrow \pm \frac{\pi }{4}$, $\mathcal{F}_{\psi }^{\mathfrak g}$ smoothly converges to a doubly covered geodesic 2-sphere with $2{\mathfrak g} + 2$ branch points; the family $\mathcal{F}_{\psi }^{\mathfrak g}$ cannot be extended in $\psi$ in the space of immersions.
3. $\mathcal{F}_{\psi }^{\mathfrak g} = \mathcal{F}_{- \psi }^{\mathfrak g}$ up to reparametrization and isometries of $\mathbb {S}^3_{\sigma }$, and accordingly the constant mean curvatures satisfy $\mathcal{H}_{\psi }^{\mathfrak g} = - \mathcal{H}_{- \psi }^{\mathfrak g}$.
4. $\mathcal{H}_{\psi }^{\mathfrak g}$ decreases strictly monotonically from zero at $\psi = 0$ to a minimum $\mathcal{H}_{\psi _m}^{\mathfrak g} = -2 \delta \sin \sigma ^{\mathfrak g}_m$ at some $\psi = \psi _{m}$, from which it increases strictly monotonically to zero for $\psi \rightarrow \frac{\pi }{4}$. The monotonicity behaviour of $\mathcal{H}_{\psi }^{\mathfrak g}$ for $\psi \in (-\frac{\pi }{4}, 0)$ is then determined by property 3.
5. The area of $\mathcal{F}_{\psi }^{\mathfrak g}$ increases strictly monotonically from $\psi = 0$ towards both $\psi = \pm \frac{\pi }{4}$ where it takes the values $A_{\pm \pi /4} = 8 \pi \delta ^{-2}$.

This theorem has the following obvious Corollary:

### Corollary 1

For every ${\mathfrak g} \gg 1$, the smooth family of conformal embeddings constructed in Theorem 2 determines a smooth MTT whose MTS sections have properties 1–5.

In Sect. 4, we note that the monotonicity of the area of the MTS sections of MTTs (holographic screens) is the key ingredient in their thermodynamic interpretation along the lines of the “second law”. We briefly discuss the area law established in [8].

## A class of spacetimes with no stable MOTSs

We start with definitions, setting out from Definition 2. We normalize the null vectors to satisfy $\langle \ell ^+, \ell ^- \rangle = -2$. Capital letters (*A*, *B*, ..) denote indices on objects on $\mathcal{F}$. The induced metric on $\mathcal{F}$ is denoted by *j* or $j_{AB}$, while its Levi-Civita derivative by *D* or $D_A$ and $\Delta _{j}:= D_A D^A$ is the Laplacian and $R_j$ the scalar curvature. The null second fundamental form of $\mathcal{F}$ with respect to $\ell ^+$ is denoted by $k^{+}$ ($k_{AB}^+$) and the torsion one form $s ~(s_A)$ is defined by

$$\begin{aligned} s (X) = - \frac{1}{2} \langle \ell ^{-}, \nabla _X \ell ^+ \rangle \quad \forall ~ \hbox {vector fields} ~X~ \hbox {on} ~ \mathcal{F}. \end{aligned}$$

### Remark 1

The motivation and inspiration for the following definition and the results on (in)stability of MOTS (cf. Sects. 3–5 of [3]) come from corresponding material on minimal surfaces of codimension 1 in manifolds with a Riemannian metric. We recall, however, that in the minimal surface case, stability can be defined via the second variation of the area functional. This is no longer the case for MOTS, which entails substantial differences (cf. Remark 2).

The key definitions for this section read as follows.

### Definition 3

*(Stability)*  We denote the Einstein tensor by $\textrm{Ein}_g = \textrm{Ric}_g - \frac{1}{2}{\mathrm R}_g g$.

- The stability operator *L* of a MOTS $\mathcal{F}$ is defined for smooth functions *w* by
  3 $$\begin{aligned} L(w) = - \Delta _{j} w + 2 s^A D_A w + \frac{1}{2} \Big (\textrm{R}_{j} - \textrm{Ein}_g(\ell ^+,\ell ^-) -2 s^A s_A + 2 D_A s^A \Big ) w \end{aligned}$$
- *L* admits a principal eigenvalue $\lambda$, which is the eigenvalue with smallest real part. This eigenvalue is necessarily real and its eigenspace is one-dimensional and spanned by an everywhere positive function $\phi$, called *principal eigenfunction*.
- The MOTS $\mathcal{F}$ is called strictly stable (resp. stable, marginally stable or unstable) provided $\lambda > 0$ ($\lambda \ge 0$, $\lambda =0$ or $\lambda <0$).

### Remark 2

The stability operator is relevant for calculating the variation $\delta _{\xi } \theta ^{+}$ of the expansion $\theta ^{+}$ with respect to an arbitrary vector field $\xi$ normal to $\mathcal{F}$. In Lemma 3.1 of [3], it was shown that

4 $$\begin{aligned} \delta _{\xi } \theta ^{+} = L(u) - \frac{1}{2} BZ \end{aligned}$$

where the functions *u*, *B* and *Z* are defined on $\mathcal{F}$ by $u:= \langle \xi , \ell ^+ \rangle$, $B:= - \langle \xi , \ell ^{-} \rangle$ and $Z:= k^{+}_{AB} k_{+}^{AB} + \textrm{Ein}_g (\ell ^+,\ell ^+)$.

The “stability operator $L_v$ in the direction of a normal vector *v*” introduced in Definition 3.1 of [3] is related to (4) as follows:

5 $$\begin{aligned} \delta _{\xi } \theta ^+ = L_v (u) ~~\hbox {for} ~ \xi = u\, v \end{aligned}$$

In particular, *L*(*u*) as defined in (3) agrees with $L_v(u)$ for $v = - \frac{1}{2}\ell ^{-}$.

The stability operator of a stable MOTS satisfies a maximum principle, cf. Lemma 4.2 of [3]:

### Lemma 1

Let $\mathcal{F}$ be a MOTS and *L* the corresponding stability operator. Let $\lambda$ be the principal eigenvalue and $\phi >0$ the principal eigenfunction. Let $\psi$ be a smooth solution of $L \psi = f$ with $f \ge 0$. Then,

(i) If $\lambda =0$, then $f =0$ and $\psi = C \phi$ for some constant *C*.
(ii) If $\lambda > 0$ and *f* is not identically zero, then $\psi >0$.
(iii) If $\lambda > 0$ and $L\psi = 0$, then $\psi =0$.

From this lemma, we can prove the following general result on the nonexistence of stable MOTS:

### Proposition 1

Let $\mathcal{F}$ be a MOTS in an n-dimensional ($n \ge 3$) spacetime $(\mathcal{M},g)$ satisfying the null convergence condition. Assume that there exists a future causal vector field $\xi$ along $\mathcal{F}$ which is not everywhere proportional to $\ell ^+$ and such that $\delta _{\xi } \theta ^{+} \ge 0$. Then, $\mathcal{F}$ cannot be strictly stable. Moreover, if $\delta _{\xi } \theta ^{+}$ is positive somewhere, then $\mathcal{F}$ cannot be marginally stable either.

### Proof

The basis $\{ \ell ^+,\ell ^-\}$ is future directed, so $\xi$ being future causal implies $u \le 0$ and $B \ge 0$. Moreover, *u* is not identically zero, because $\xi$ is not everywhere proportional to $\ell ^+$. Together with Equ. (4) and the hypotheses of the proposition, this implies $L(u) \ge 0$. If $\mathcal{F}$ is strictly stable (i.e. $\lambda >0)$, items (ii) and (iii) of Lemma 1 imply that *u* is either strictly positive or identically zero, which is a contradiction. This proves the first claim of the proposition. Finally, if $\mathcal{F}$ is marginally stable, then item (i) of the Lemma yields $\delta _{\xi } \theta ^{+} + \frac{1}{2}B Z =0$, which cannot happen if $\delta _{\xi } \theta ^{+}$ is positive somewhere. $\square$

We are now ready to prove Theorem 1 stated in the Introduction. We use Greek indices on spacetime objects and sum over repeatedly occurring ones.

### Proof of Theorem 1

In Corollary 1 of [10], the following general identity is proved for variations of $\theta ^{+}$ of a MOTS along an arbitrary vector field $\xi$ in terms of the deformation tensor $a(\xi ):= \pounds _{\xi } g$ of $\xi$

6 $$\begin{aligned} \delta _{\xi } \theta ^{+} = -\frac{1}{4} \theta ^{-} a(\xi )_{\alpha \beta } \ell ^{\alpha }_+ \ell ^{\beta }_+ - a(\xi )_{\alpha \beta } e_A^{\alpha } e_B ^{\beta } k_{+}^{AB} + j^{AB} e_A^{\alpha } e_B^{\beta } \ell ^{\nu }_+ \left( \frac{1}{2} \nabla _{\nu } a(\xi )_{\alpha \beta } - \nabla _{\alpha } a(\xi )_{\beta \nu } \right) \end{aligned}$$

where $\{e^{\alpha }_A\}$ is any basis of tangent vectors to $\mathcal{F}$. Assume now that $\xi$ is a conformal Killing vector, so that $a(\xi ) = 2 \Psi g$. Inserting into (6) yields

$$\begin{aligned} \delta _{\xi } \theta ^{+} = j^{AB} e_A^{\alpha } e_B^{\beta } \ell ^{\nu }_+ \left( \nabla _{\nu } \Psi g_{\alpha \beta } - 2 \nabla _{\alpha } \Psi g_{\beta \nu } \right) = (n-2) \ell ^{\nu }_+ \nabla _{\nu } \Psi . \end{aligned}$$

Since $\ell ^+$ is future directed and $\Psi$ is a temporal function, we have $\delta _{\xi } \theta ^{+} >0$, and the result is a consequence of Proposition 1. $\square$

### Remark 3

Recall that MOTS were defined in Definition 2 by the vanishing of (at least) one of the null expansions. As the “outer” amendment apparently plays no role, neither in that Definition nor in the discussion above, the simpler notion “marginal surface” (introduced by Hayward [18]) would suffice. To indicate some benefit of the “MOTS” terminology, we recall that in Definition 3.1 of [3], stability of the MOTS with respect to any direction *v* was defined by requiring that the operator $L_v(w)$ (Eq. (5)) has nonnegative lowest eigenvalue. This is equivalent to nonnegativity of either side of (5) provided $\ell ^+$ points in the same direction as $\xi = u\,v$, viz. $u = \langle \xi , \ell ^{+} \rangle \ge 0$. Calling these directions “outer” (or “inner”) is just more efficient terminology than some “... points in the same (or opposite) direction...” amendment. The next section provides corresponding “unstable examples”.

### Example 1

(*The Friedman–Lemaitre–Robinson–Walker Universe* (*FLRW*)) Before focusing on dS in the next section, we apply Theorem 1 to n-dim. FLRW, viz.

7 $$\begin{aligned} ds^2 = -dt^2 + a(t)^2 \left( \frac{dr^2}{1 - \kappa r^2} + r^2 d\Omega ^2 \right) \end{aligned}$$

where $d\Omega ^2$ is the spherical metric in $n-2$ dimensions and $\kappa \in \{-1,0,1\}$.

The requirements that $\Psi$ is a temporal function for the conformal Killing field $\xi =a \partial _t$ and the null convergence condition translate into the following conditions on the scale factor *a*(*t*), respectively

8 $$\begin{aligned} \ddot{a} >0, \qquad \ddot{a} \le \frac{\dot{a}^2 + \kappa }{a}. \end{aligned}$$

Here, dot is the derivative with respect to *t*. If conditions (8) hold, all MOTS are unstable.

We remark in this context that MOTS in FLRW was investigated, e.g., in [16, 20]. In the latter paper, it was found that in closed ($\kappa = 1$) 4-dim. FLRW, a certain family of MOTS called CMC Clifford tori are always unstable. In the next section, we will analyse systematically families of CMC-MOTS in umbilic slices of dS.

## MOTSs and MOTTs in de Sitter spacetime

### Instability of MOTS

The following easy consequence of Theorem 1 was already sketched in the Introduction.

#### Corollary 2

All MOTS in de Sitter spacetime are unstable.

#### Proof

In terms of the coordinates (1), $\xi = \frac{\partial }{\partial \sigma }$ is a future directed, timelike conformal Killing vector field satisfying

$$\begin{aligned} \pounds _{\xi } g_{\tiny {\text {dS}}} = 2 \tan \sigma \, g_{\tiny {\text {dS}}}. \end{aligned}$$

Since $\tan$ is an increasing function and $\sigma$ is a temporal function, so is $\Psi = \tan \sigma$. Hence, Theorem 1 implies the claim. $\square$

#### Remark 4

There is a certain analogy between de Sitter spacetime on which we focus in this section and round spheres, in the sense that both are maximally symmetric spaces of constant positive curvature. Recalling also the analogy between the stability definitions for minimal surfaces and MOTS (cf. Remark 1), Corollary 2 can be seen as a counterpart to a result of [23] (namely case $p = n-1$ of Thm 5.1.1) that all minimal surfaces of codimension 1 on the round ${\mathbb S}^n$ are unstable. However, there is no obvious analogy between the proofs, and we do not make any attempt of establishing one here.

### MOTS in umbilic slicings

From now on, we restrict ourselves to dS in 3+1 dimensions. Our aim is to locate MOTTs in the three umbilic and constant curvature slicings of de Sitter, namely flat and hyperboloidal and spherical. Our focus will be on the latter case.

#### Preliminaries

We consider a spacelike hypersurface $(\mathcal{N}, h, K)$ with metric *h*, scalar $\hbox {curvature}^{\,3}\,R$, covariant $\hbox {derivative}^{\,3}\nabla$, extrinsic curvature *K* (w.r.t. a future-pointing unit normal *N*) and mean curvature $tr_h K$ (i.e. again with “physics convention”, cf. Definition 1). The constraints take the form

9 $$\begin{aligned} R_h - |K|_h^2 + (tr_h K)^2= &   6 \delta ^2 \end{aligned}$$

10 $$\begin{aligned} div_h \left( K - (tr_h K) h \right)= &   0. \end{aligned}$$

##### Lemma 2

Let $(\mathcal{N}, h,K)$ be an umbilic slice in de Sitter, i.e. the extrinsic curvature satisfies $K = \beta h$ for some function $\beta$. Then, $\beta$ is constant on $\mathcal{N}$. Furthermore, any MOTS $\mathcal{F} \subset \mathcal{N}$ with $\theta ^{+} = 0$ is a MTS and CMC as well, with mean curvature $H = div_{h} X = - 2 \beta$ where *X* is a unit outward normal vector to $\mathcal{F}$ in $\mathcal{N}$, i.e. $\langle X, \ell ^+ \rangle > 0$.

##### Proof

The constancy of $\beta$ follows from the constraint (10). For the second statement, we use the normalization $\langle \ell ^+, \ell ^- \rangle = -2$ and the decomposition $\ell ^{\pm } = N \pm X$. This induces the following decomposition of $\theta ^{\pm }$

11 $$\begin{aligned} \theta ^{\pm } = \pm H + tr_{j} K =\pm H + 2 \beta \end{aligned}$$

which implies the assertion, with $\theta ^{-} = 4 \beta$ on the MOTS $\theta ^{+} = 0$. $\square$

##### Remark 5

In the following subsections, we consider as examples umbilic slicings $(\mathcal{N}, h,K)$ of constant curvature (i.e. *h* is flat, spherical or hyperboloidal) in dS. In this special situation, a recent result of [7] implies instability of all MOTS $\mathcal{F}$ contained in such slices. The argument goes as follows. The spaces $\mathcal{N}$ in question are homogeneous in the sense that their isometry groups act transitively. In particular, for a given MOTS $\mathcal{F}$, any pair of points $p \in \mathcal{F}$ and $q \not \in \mathcal{F}$ can be joined by a group element $\rho$, i.e. $q = \rho (p)$. Hence, the Killing vector $\xi$ of $\mathcal{N}$ tangent to this orbit cannot be everywhere tangent to $\mathcal{F}$. Now by Theorem 1.6 of [7] or by Theorem 8.1 of [3], $\mathcal{F}$ is either unstable or marginally stable, with $\xi$ nowhere tangent to $\mathcal{F}$ in the latter case. But this case is ruled out by Theorem 5.1 of [7], since by Lemma 2, $H = -2 \beta =$ const. on an umbilic slice.

While the results of [7] also cover other situations than the aforementioned one (as discussed in that paper), there is not much further overlap with the general settings considered in Proposition 1 and Corollary 2. Of course, the latter results apply to umbilic slices.

These slicings are conveniently defined in terms of the dS hyperboloid

12 $$\begin{aligned} -x_0^2 + x_{\alpha } x_{\alpha } = \delta ^{-2} \qquad \alpha ,\beta \in \{ 1,2,3,4\}. \end{aligned}$$

embedded in 5d Minkowski space

13 $$\begin{aligned} ds^2 = -dx_0^2 + dx_{\alpha } dx_{\alpha } \end{aligned}$$

Above and henceforth, repeated indices are summed over. A useful reference for the slicings considered is Sect. 4 of [17].

#### Flat slicing

In terms of the coordinate transformation

14 $$\begin{aligned} x_0= &   \delta ^{-1} \sinh \delta t + \frac{\delta }{2} r^2 e^{\delta t} \end{aligned}$$

15 $$\begin{aligned} x_1= &   \delta ^{-1} \cosh \delta t - \frac{\delta }{2} r^2 e^{\delta t} \end{aligned}$$

16 $$\begin{aligned} x_i= &   e^{\delta t} y_i \qquad (i \in \{2,3,4\}) \qquad r^2 = y_iy_i \end{aligned}$$

and upon introducing polar coordinates on $\mathbb {S}^2$, the induced metric on the hyperboloid (12) reads

17 $$\begin{aligned} ds^2 = -dt^ 2 + e^{2 \delta t} [dr^2 + r^2(d\vartheta ^2 + \sin ^2 \vartheta d\phi ^2)] \quad r \in [0,\infty ),~ \vartheta \in [0,\pi ], ~\phi \in [0,2\pi ] \end{aligned}$$

Clearly, the $$t = $$const. [$$t \in (-\infty , \infty )$$] slicing is flat and umbilic (with $$\beta = \delta $$) but covers only half of dS as described by (1).

In order to determine the MOTS in these slices, we recall that closed CMC surfaces embedded in flat 3-space must necessarily be round spheres [2].

A sphere of radius $r=R$ in a slice $t= T$ obviously has the induced metric

18 $$\begin{aligned} ds_R ^2 = e^{2 \delta T} R^2(d\vartheta ^2 + \sin ^2 \vartheta d\phi ^2). \end{aligned}$$

We now choose the counter-intuitive label *“outgoing”* for the normal vector *X* pointing towards *decreasing* *r*, viz.:

19 $$\begin{aligned} X = - e^{ - \delta T} \frac{\partial }{\partial r}. \end{aligned}$$

We find for the mean curvature w.r.t. *X*

20 $$\begin{aligned} H = div_h X= - \frac{2}{R} e^{-\delta T}. \end{aligned}$$

It is only with this choice that (11) can be solved to determine the location of a marginally *outer* trapped tube with MOTS sections $\theta ^+ = 0$, namely:

21 $$\begin{aligned} e^{-\delta T} = \delta R \end{aligned}$$

in consistency with Definition 2. We note that $H = - 2\delta = const.$ along the MOTT.

#### Hyperboloidal slicing

We perform the coordinate transformation

22 $$\begin{aligned} x_0= &   \delta ^{-1} \sinh \delta t \cosh r \end{aligned}$$

23 $$\begin{aligned} x_1= &   \delta ^{-1} \cosh \delta t \end{aligned}$$

24 $$\begin{aligned} x_i= &   \delta ^{-1} z_i \sinh \delta t \sinh r \qquad (i \in \{2,3,4\}) \qquad z_iz_i = 1 \end{aligned}$$

which yields for the induced metric on the hyperboloid (12)

25 $$\begin{aligned} ds^2 = -dt^2 + \delta ^{-2} \sinh ^2 (\delta t) [dr^2 + \sinh ^2 r (d\vartheta ^2 + \sin ^2 \vartheta d\phi ^2)] \end{aligned}$$

where $t\in (-\infty ,\infty )$, $r \in [0, \infty )$ and $\vartheta \in [0,\pi ], ~\phi \in [0,2\pi )$. This slicing is umbilic with $\beta = \delta \coth \delta T$ for $t = T =$const.; again it covers only part of dS given by (1), cf. Section 4.4.2 of [17].

Here, we restrict ourselves to calculating round CMC spheres; as to more general shapes cf. Remark 6. Then, the discussion becomes quite similar to the flat case of the previous subsection. The induced metric on slices $$t = T =$$const., $$ r =R =$$const. reads

$$\begin{aligned} ds_R^2 = \delta ^{-2} \sinh ^2 (\delta T) \sinh ^2 R (d\vartheta ^2 + \sin ^2 \vartheta d\phi ^2) \end{aligned}$$

We now take as *outgoing* unit normal vector to the $R= const$ slices

$$\begin{aligned} X = - \delta \sinh ^{-1} (\delta T) \frac{\partial }{\partial r} \end{aligned}$$

which yields for the mean curvatures of the MOTSs,

26 $$\begin{aligned} H = div_h X= - 2 \delta \sinh ^{-1} (\delta T) \coth R. \end{aligned}$$

It then follows from (11) that the MOTT with spherical sections are determined by the equation

27 $$\begin{aligned} \cosh \delta T = \coth R . \end{aligned}$$

For the mean curvatures of the MOTS sections of these MOTTs, we obtain

28 $$\begin{aligned} H = -2 \delta \cosh R \le -2 \delta \end{aligned}$$

##### Remark 6

By the maximum principle, the mean curvature of a closed CMC surface embedded in the unit hyperboloid $\mathbb {H}^3$ must satisfy either $H \ge 0$ or $H \le -2$ (cf. e.g. [13]), in consistency with (28). We also recall the “Lawson correspondence” [13, 19], which is a locally bijective relation between CMC surfaces in 3-dim space forms. In particular, CMC surfaces in the unit hyperboloid $\mathbb {H}^3$ with $H \le -2$ have corresponding CMC or minimal surfaces in flat space or in the unit sphere $\mathbb {S}^3$. We restrict the detailed discussion to the latter case.

#### Complete spherical slicing

Introducing the coordinates

29 $$\begin{aligned} x_0= &   \delta ^{-1} \sinh \delta t \end{aligned}$$

30 $$\begin{aligned} x_\alpha= &   \delta ^{-1} z_{\alpha } \cosh \delta t \qquad (\alpha \in \{1, 2,3,4\}) \qquad z_{\alpha }z_{\alpha } = 1, \end{aligned}$$

we obtain

31 $$\begin{aligned} ds^2 = -dt^2 + \delta ^{-2} \cosh ^2 (\delta t) [d\tau ^2 + \sin ^2 \tau (d\vartheta ^2 + \sin ^2 \vartheta d\phi ^2)] \qquad \tau \in [0, \pi ] \end{aligned}$$

This is equivalent to (1) where the time coordinates are related by $\tan \frac{\sigma }{2} = \tanh \frac{\delta t}{2}$. We proceed the discussion in terms of (1).

The extrinsic and mean curvatures of the surfaces $\sigma = const$ read

32 $$\begin{aligned} K = (\delta \sin \sigma ) \, h \qquad tr_h K = 3 \delta \sin \sigma . \end{aligned}$$

Therefore, $\beta = \delta \sin \sigma$ in the notation of Lemma 2. In contrast to the previous slicings Sects. 3.2.2 and 3.2.3, we can now solve (11) for either sign which yields

33 $$\begin{aligned} H = \mp 2 \delta \sin \sigma . \end{aligned}$$

**3.2.4.1 Spherical MOTS**

In this and subsection 3.2.4.2, our presentation largely follows [5, Sect. 5] and [9, Ch. 5].

In polar coordinates, (1) takes the form

34 $$\begin{aligned} ds^2 = \delta ^{-2}\cos ^{-2}\sigma \left[ d\tau ^ 2 + \sin ^2 \tau \left( d \vartheta ^2 + \sin ^ 2 \vartheta d\varphi ^2 \right) \right] ; ~~\tau , \vartheta \in [0, \pi ], \varphi \in [0, 2\pi ) \end{aligned}$$

For round 2-spheres $\tau = const.$, we obtain from (11):

35 $$\begin{aligned} \theta ^{\pm } = 2 \delta [\pm \cos \sigma \cot \tau + \sin \sigma ]. \end{aligned}$$

The MOTSs given by $\tau _0^{\pm } = \pm \sigma _0 + \frac{\pi }{2}$ are spheres with radius $\delta ^{-1}$.

**Fig. 1:**
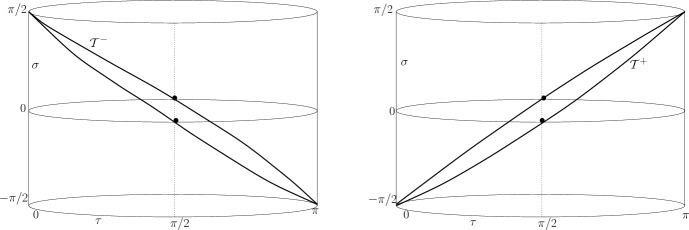
MOTTs $\mathcal{T}^-$ (left) and $\mathcal{T}^+$ (right) with spherical CMC sections

Figure 1 shows, via a conformal rescaling (“Einstein cylinder”, conformal factor $\delta ^{-2} \cos ^{-2}\sigma$), the MOTTs $\mathcal{T}^{\pm }$ determined by the two families of MOTSs. $\mathcal{T}^{\pm }$ are null surfaces. The $\varphi$ and $\vartheta$ directions are suppressed; hence, spheres reduce to two points; the dots at $\sigma = 0$ correspond to the equators on $\mathbb {S}^3$, while the conical convergence towards $\sigma = \pm \frac{\pi }{2}$ is due to the conformal factor. The drawing is sketchy (i.e. not a faithful result of calculation).

**3.2.4.2 Toroidal MOTS**

We recall the toroidal foliation of $\mathbb {S}^3$ in terms of the following coordinates:

36 $$\begin{aligned} ds^2 = \delta ^{-2}\cos ^{-2} \sigma (d \tau ^2 + \sin ^2 \tau \, d \gamma ^2 + \cos ^2 \tau \,d\xi ^2 ); \quad \tau \in [0, \frac{\pi }{2}], \quad \gamma , \xi \in [0, 2\pi ) \end{aligned}$$

On a torus $\mathcal{F}$ given by $\tau = const.$, we find from (11):

37 $$\begin{aligned} \theta ^{\pm } = 2 \delta [\pm \cos \sigma \cot 2 \tau + \sin \sigma ] \end{aligned}$$

which formally differs just by a factor of 2 in the $\cot$-term from the spherical case (35). This entails that the MOTSs determined by $\theta ^{\pm } = 0$ and given via $\tau = \tau ^{\pm }$ on some slice $\sigma = \sigma _0$ are now tori located at $\tau _0^{\pm }= \pm \frac{\sigma _0}{2} + \frac{\pi }{4}$. As above, the MOTS exist for all $\sigma \in (- \frac{\pi }{2}, \frac{\pi }{2})$ but *now they form timelike MOTTs.* The area of its toroidal sections

38 $$\begin{aligned} \frac{A}{4\pi } = \vert \frac{\sin \tau \cos \tau }{\delta ^2 \cos ^2 \sigma } \vert = \vert \frac{\sin 2 \tau }{2 \delta ^2 \cos ^2 \sigma } \vert =\vert \frac{\sin (\pm \sigma + \pi /2) }{2 \delta ^2 \cos ^2 \sigma } \vert = \frac{1}{2 \delta ^2 \cos \sigma } \end{aligned}$$

diverges when $\sigma \rightarrow \pm \frac{\pi }{2}$ and approaches the minimum $\frac{1}{2 \delta ^2}$ at $\sigma = 0$.

**Fig. 2:**
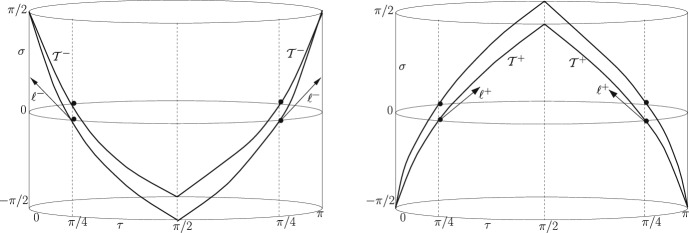
MOTTs $\mathcal{T}^-$ (left) and $\mathcal{T}^+$ (right) with toroidal CMC sections

Figure 2 shows again the Einstein cylinder with two dimensions suppressed; each MOTS $\mathcal{T}^{\pm }$ reduces to 4 points; the Clifford torus is indicated by 4 dots at $\sigma = 0$. The MOTSs are timelike, which is visualized by the null vectors $\ell ^{\pm }$. As before, this figure is also sketchy.

**3.2.4.3 MOTS of higher genus**

We now turn to MOTSs and MOTTs of higher genus ${\mathfrak g}$. A natural strategy is to set out from a minimal surface at the time-symmetric slice $\sigma = 0$, as such surfaces also have played special roles in the cases of genus 0 and 1. We would like to show existence of their evolution to MOTTs, and determine the causal character of the latter as well as the evolution of the area of their MOTS sections. Unfortunately, for genus ${\mathfrak g} > 1$, explicit expressions for the embedding functions and other geometric quantities seem not to be available. Moreover, instability of the MOTS prevents us from applying the general results of [3, 4].

Following [11], we adopt here the following strategy. It is known that any CMC surface in $\mathbb {R}^3$ or $\mathbb {S}^3$ gives a solution to a certain Lax pair; vice versa, one can construct CMC surfaces as solutions to certain Lax pairs using the Sym-Bobenko formula. Making use of this, the DPW method [14] is a way of constructing CMC surfaces from holomorphic potentials on Riemann surfaces by solving a certain Cauchy problem. To ensure that the resulting CMC surface is well-defined, one has to solve in addition the so-called monodromy problem. In this way, the authors of [11] construct families of CMC embeddings on $\mathbb {S}^3$ for every sufficiently high genus ${\mathfrak g}$; the latter restriction comes from the use of an implicit function theorem argument near $t = 0$, where $t = \frac{1}{2{\mathfrak g}+2} \mathcal{K}^{-1/2}$, and the quantity $\mathcal{K}$ arises in the monodromy construction, cf. Prop. 9 of [11].

We present in Theorem 3 below a combination of Theorem 1 and a part of Theorem 2 of [11]; item 4. also contains a slight extension of the aforementioned results. Moreover, we change the parametrisation from $\varphi$ to $\psi = \varphi - \frac{\pi }{4}$ which simplifies the presentation.

##### Theorem 3

(Theorems 1 and 2 of [11]) Our setting is the 3-dim. unit sphere $\mathbb {S}^3$. For every ${\mathfrak g} \in \mathbb {N}$ sufficiently large, there exists a smooth family of conformal CMC embeddings $f_{\psi }^{\mathfrak g}: \mathcal{R}^{\mathfrak g} \rightarrow \mathbb {S}^3$ from a Riemann surface with genus *g* and parameter $\psi \in (-\frac{\pi }{4}, \frac{\pi }{4})$ satisfying

1. $f_{0}^{\mathfrak g}$ is the Lawson surface $\xi _{1, {\mathfrak g}}$ of genus ${\mathfrak g}$.
2. For $\psi \rightarrow \pm \frac{\pi }{4}$, the embedding $f_{\psi }^{\mathfrak g}$ smoothly converges to a doubly covered geodesic 2-sphere with $2{\mathfrak g} + 2$ branch points, i.e. the family $f_{\psi }^{\mathfrak g}$ cannot be extended in the parameter $\psi$ in the space of immersions.
3. $f_{\psi }^{\mathfrak g} = f_{- \psi }^{\mathfrak g}$ up to reparametrization and (orientation reversing) isometries of $\mathbb {S}^3$, and accordingly, the constant mean curvatures satisfy $H_{\psi }^{\mathfrak g} = -H_{-\psi }^{\mathfrak g}$.
4. The mean curvature $H_{\psi }^{\mathfrak g}$ of $f_{\psi }^{\mathfrak g}$ decreases strictly monotonically from zero at $\psi = 0$ to some minimal value $H_{\psi _m}^{\mathfrak g}$ for $\psi = \psi _m$ from which it increases strictly monotonically to zero for $\psi \rightarrow \frac{\pi }{4}$. The monotonicity behaviour for $\psi \in (-\frac{\pi }{4},0)$ then follows from property 3.
5. The Willmore energy Equ. (2) of $f_{\psi }^{\mathfrak g}$ increases strictly monotonically from $\psi = 0$ towards both $\psi \rightarrow \pm \frac{\pi }{4}$ where $W_{\pm \pi /4} = 8 \pi$.

##### Proof

Items 1–3 and 5 follow as in the proofs of Thms. 1 and 2 of [11]; to show item 4, we note that from Proposition 34 of [11], the mean curvature $H^{\mathfrak g}_{\psi } = H(t({\mathfrak g}), \psi )$ with $t ({\mathfrak g})= \frac{1}{2{\mathfrak g} + 2} \mathcal{K}^{-1/2}(t,\psi )$ is smooth and its Taylor expansion in *t* takes the form

39 $$\begin{aligned} H(t,\psi ) = 4 t \cos 2\psi \ln \left[ \tan (\psi + \frac{\pi }{4})\right] + O(t^2). \end{aligned}$$

Here, $\mathcal{K}$ depends on *t* itself but such that $\mathcal{K}(t,\psi ) = 1 + O(t^2)$ near $t = 0$, cf. Sect. 6.3 of [11]. A calculation shows that at the values $\psi = \psi _0$ and $\psi = -\psi _0$, $H_0 (\psi )= (\partial H/\partial t)(0,\psi )$ takes on its unique non-degenerate minimum and maximum, respectively, i.e.

40 $$\begin{aligned} \frac{dH_0}{d\psi }(\pm \psi _0) = 0 \qquad \frac{d^2H_0}{d\psi ^2} (\pm \psi _0) \ne 0, \end{aligned}$$

where $\pm \psi _0$ are given implicitly by

41 $$\begin{aligned} (\sin 2 \psi _0) \ln \left[ \tan \left( \psi _0 + \frac{\pi }{4}\right) \right] = 1. \end{aligned}$$

Moreover, there hold the monotonicity properties

42 $$\begin{aligned} \frac{dH_0}{d\psi }> &   0 \qquad \forall ~ \psi \in \left[ \left. - \frac{\pi }{4}, -\psi _0\right) \right. ~ \hbox {and}~ \psi \in \left. \left( \psi _0, \frac{\pi }{4}\right. \right] \end{aligned}$$

43 $$\begin{aligned} \frac{dH_0}{d\psi }< &   0 \qquad \forall ~ \psi \in ( -\psi _0, \psi _0) \end{aligned}$$

We now observe that there exists a $\psi _m(\psi , t) = \psi _0 + O(t)$ such that, for sufficiently small *t*, properties (40), (42) and (43) hold for $H(t,\psi )$ as well, with $\psi _0$ replaced by $\psi _m$ everywhere. Away from the zeros of *H* which are at $\psi = 0,\pm \frac{\pi }{4}$ (cf. Prop. 18 of [11]), this follows from smoothness and from the non-degeneracy (40), while in a neighbourhood of the zeros of *H*, the assertion holds by virtue of Prop. 34 of [11]. $\square$

##### Remark 7

The convergence properties of the CMC surfaces for $\psi \rightarrow \pm \frac{\pi }{4}$ (point 2 of Theorem 3) are explained in Prop. 26 of [11].

We are now ready to prove Theorem 2 stated in the Introduction.

##### Proof of Theorem 2

We define the conformal rescaling $\mathcal{I}_{\sigma }: \mathbb {S}^3 \rightarrow \mathbb {S}^3_{\sigma }$ from the unit sphere to the sphere of radius $\rho = \delta ^{-1} \cos ^{-1} \sigma$. This clearly induces a scaling for all embedded surfaces; we note that the mean curvature of such surfaces changes from *H* to $\mathcal{H}_{\sigma } = \delta H \cos \sigma$. We now define the embedding $\mathcal{F}_{\psi }^{\mathfrak g}: \mathcal{R}^{\mathfrak g} \rightarrow \mathbb {S}^3_{\sigma }$ by $\mathcal{F}_{\psi }^{\mathfrak g} = \mathcal{I}_{\sigma (\psi )} \circ f_{\psi }^{\mathfrak g}$ where $\sigma (\psi )$ is constructed as follows. We recall from (33) that any CMC surface with mean curvature $\mathcal{H}_{\sigma }$ on $\sigma =$ const. corresponds to a MTS if $\mathcal{H}_{\sigma } = \mp 2 \delta \sin \sigma$. In particular, if the mean curvatures $H_{\psi }^{\mathfrak g}$ of the CMC surfaces $f_{\psi }^{\mathfrak g}$ constructed in Theorem 3 satisfy

44 $$\begin{aligned} H_{\psi }^{\mathfrak g} = \frac{\mathcal{H}_{\sigma }}{\delta \cos \sigma } = \mp 2 \tan \sigma , \end{aligned}$$

they build up MTTs, which serves to define $\sigma ({\psi })$.

In order to prove the final statement 5. on monotonicity of the area, we show that the Willmore functional ${W}_{\sigma }$ (2) built from the rescaled quantities is just the area of the embedded MOTS. To see this, we note that at time $\sigma$, the rescaled area is $\mathcal{A}_{\sigma } = A \delta ^{-2} \cos ^{-2} \sigma$, while the rescaled mean curvature is $\mathcal{H}_{\sigma } = \delta H \cos \sigma$. Inserting in (2), we obtain $W_{\sigma } = \delta ^2 A_{\sigma }$ which finishes the proof. $\square$

##### Remark 8

It is stated in Theorem 1 in [11] that “the moduli space of genus ${\mathfrak g}$ CMC surfaces is one-dimensional at the Lawson surface $\xi _{1,\mathfrak g}$”. This is not to be understood in the sense that the MTTs constructed in Theorem 2 are unique, as also indicated in point 3. of that Theorem. In fact, isometries of $\mathbb S^3$ which are not tangent to $\mathcal{F}_{\psi }^{\mathfrak g}$ (and which exist, cf. Remark 5) will “move around” $\mathcal{F}_{\psi }^{\mathfrak g}$ at any parameter value $\psi$.

##### Remark 9

We recall that there are no non-trivial isometries of $\mathcal{F}_{\psi }^{\mathfrak g}$ in the case ${\mathfrak g} \ge 2$ in which the Euler number $\chi = 2(1-\mathfrak g)$ is negative. Assuming the contrary, the Poincaré–Hopf theorem implies that $\chi$ is also the sum of all indices of the (isolated) zeros of the corresponding Killing field. But if an isometry in 2 dim. has fixed points, it is necessarily a rotation in a neighbourhood. Hence, all Killing indices are $+1$ which implies that $\chi$ is nonnegative. This restricts the topology of $\mathcal{F}_{\psi }^{\mathfrak g}$ to the sphere or the torus if it has isometries.

##### Remark 10

We note that the conformal rescaling $\mathcal{I}_{\sigma }: \mathbb {S}^3 \rightarrow \mathbb {S}^3_{\sigma }$ involved in passing from Theorems 3 to 2 is precisely the inverse of the one used to construct the Penrose diagrams in Figs. 1 and 2 of dS. Hence, a Penrose-type diagram arises just by directly “stacking” the family of CMC surfaces $f_{\psi }^{\mathfrak g}$ constructed in Theorem 3. We recall, however, that the embedding parameter $\psi$ is not a monotonic function of the cosmological time $\sigma$—rather, the constructed MOTT “turns around” at $\psi = \pm \psi _m$.

## Discussion

Note that monotonicity of area holds along MTTs with MTS sections of spherical, toroidal and high-genus topology in the complete spherical slicing of dS as described in Sect. 3.2.4. Except for spherical MTS whose area stays constant, the area increase is even strictly monotonic upon moving away from the time-symmetric surface. In view of the extensively discussed correspondence between area and entropy, our MTTs are thus candidates for satisfying the “second law” of thermodynamics in an appropriate setting.

Such settings arise in attempts of understanding the “information paradox” of black holes, and in attempts of making sense of the idea of AdS/CFT correspondence (cf. e.g. [15] and references therein). In the latter context, MTTs run under the name “holographic screens”. In fact an area theorem has been obtained in [8] (cf. Theorem IV.3) and [1] for so-called “regular holographic screens” whose definition consists of four somewhat subtle conditions (cf. Definition II.8 of [8]). We have not been able to extract the required information from our Theorem 2 to check these requirements; in particular, the causal character of the constructed MTT is unclear except that they must be spacelike near the turning points $\pm \psi _m$ where the mean curvatures achieve their extrema $\pm \mathcal{H}^{\mathfrak g}_{\psi _m}$ (cf. item 4 of Theorem 2). In order to obtain monotonicity of area of the MTS (item 5 of Theorem 2), we are thus bound to the argument involving their rescaled Willmore energy (item 5 of Theorem 3).

## Data Availability

No new data were created or analysed in this paper.
